# Rifampicin and ofloxacin dual therapy as an adjunct to standard MB-MDT in the management of nodulo-ulcerative lepromatous leprosy with histoid-like features: a case report

**DOI:** 10.3389/fmed.2025.1596617

**Published:** 2025-08-25

**Authors:** Varun H, Adarshlata Singh, Bhushan Madke, Anurag Mittal

**Affiliations:** Department of Dermatology, Venereology and Leprosy, Datta Meghe Institute of Higher Education and Research, Wardha, India

**Keywords:** leprosy, Hansen’s disease, lepromatous leprosy, novel treatment, treatment regime, histoid Hansen, neglected tropical disease

## Abstract

Leprosy, induced by *Mycobacterium leprae*, and in some cases, *Mycobacterium lepromatosis*, remains an important public health issue in endemic regions despite ongoing elimination efforts. Histoid Hansen’s disease, a variant of lepromatous leprosy, is characterised by shiny, well-defined nodules and a heavy acid-fast bacillary load. We present a case of a 50-year-old male agricultural worker from rural central India presenting during a community health camp with multiple cutaneous nodules clinically suggestive of histoid leprosy. Slit-skin-smears (SSS) revealed abundant needle-shaped acid-fast bacilli, with numerous globi, whereas histopathological examination of a lesional biopsy supported the diagnosis of lepromatous leprosy (LL). This report presents a novel two-step treatment approach that is aimed at rapidly reducing infectivity using a 28-day course of rifampicin and ofloxacin (RO therapy), followed by initiation of multi-bacillary multidrug therapy (MB-MDT) to ensure sustained bacillary clearance. Repeat SSS demonstrated fragmented and granular acid-fast bacilli, indicating effective bactericidal activity. This case underscores the need for vigilant surveillance, early identification, and potential strategies to combat relapses and drug-resistant strains in the post-elimination era.

## Introduction

Leprosy is a chronic granulomatous infection caused by *Mycobacterium leprae* and, less commonly, *Mycobacterium lepromatosis*, remains a World Health Organisation (WHO)-designated neglected tropical disease (NTD) (1). Its clinical spectrum ranges from a solitary hypopigmented, hypo-aesthetic patch to disfiguring nodular and infiltrative forms. Although significant progress has been made under the Indian National Leprosy Eradication Programme (NELP), India continually contributes to a substantial proportion of global cases (2).

Histoid Hansen’s disease is a rare variant of LL characterised by nodular, well-demarcated shiny lesions–termed histoid lepromas–and a unique histological profile with spindle-shaped histiocytes alongside a heavy bacillary load comprising long, slender, needle-like bacilli. These bacilli typically form ill-defined clumps, with globi observed on SSS only in rare cases, likely due to defective glial substance production that normally facilitates globi formation (3–6). It is also a well-documented fact that histoid lepromas are more prone to form in drug-resistant strains (7–9).

This report describes a case of nodulo-ulcerative histoid-like lepromas in an adult male whose clinical presentation aligned with histoid leprosy, while SSS revealed overlapping features and histopathology diagnosing LL. The patient was treated with a novel two-step treatment strategy–employing an initial fixed drug combination (FDC) of daily rifampicin 600 mg and ofloxacin 400 mg to rapidly reduce the bacillary load and transmissibility, followed by standard 12-month MB-MDT for definitive management aimed at eliminating persisters.

## Case description

A 50-year-old male agricultural worker presented to a two-day diagnostic health camp organised in a rural area in central India with a six-month history of multiple raised lesions distributed all across the body. They were insidious in onset and gradually progressed to involve both ears, face, trunk and lower limbs over 5 months. The patient also reported occasional epistaxis, tingling and numbness of extremities, and a history of slipping footwear, suggesting peripheral nerve impairment.

**Figure 1:**
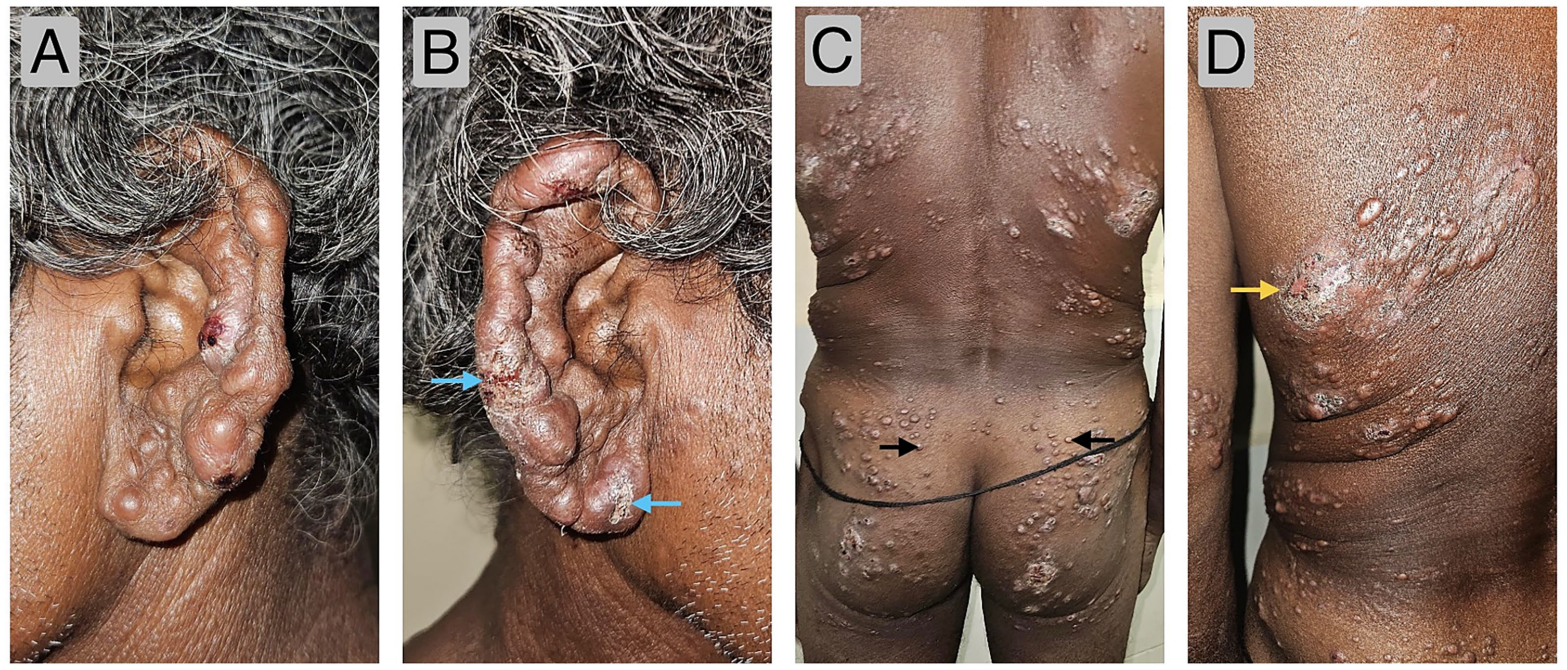
**(A,B)** Clinical images of the bilateral external ear showing papulonodular thickening of the ear helices, anti-helices and pinnae along with “rat-bite” ulcers over the helices and left ear lobe (blue arrows). **(C)** Clinical image showing multiple, well-defined, shiny nodules distributed over the back and both buttocks (black arrows). **(D)** Clinical image showing an ill-defined, erythematous plaque with erosion and crusting on the left mid-back (yellow arrow).

Cutaneous examination revealed multiple, well-defined, skin-coloured, shiny keloidiform nodules–some with central umbilications, erosions, ulcers and crusting–distributed across the face, upper limbs, trunk and lower limbs (Figure 1). Close inspection of the ears demonstrated papulo-nodular infiltration of the pinnae, antihelices, and earlobes and two “rat-bitten” ulcers (Figures 1A,B), with shiny nodules over the supraorbital areas and madarosis of the eyebrows. Peripheral nerve examination revealed cord-like thickening of the bilateral ulnar, radial cutaneous, median and common peroneal nerves with a glove-and-stocking pattern of peripheral sensory loss and grade III motor weakness in the small muscles of the hands. The patient had not sought medical attention prior to his presentation.

**Figure 2:**
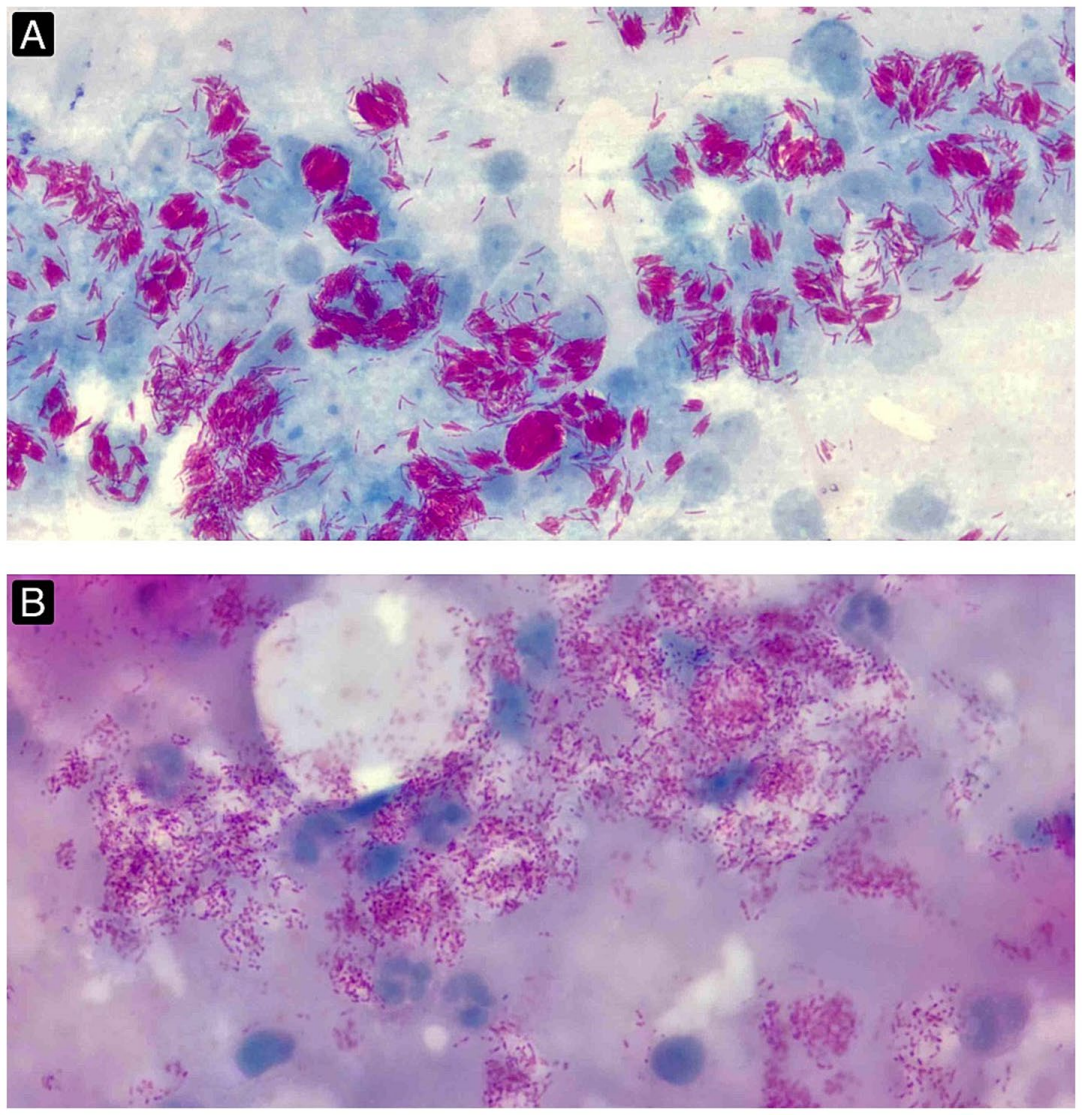
**(A)** SSS stained using the modified ZN method, demonstrating multiple clusters of acid-fast bacilli and globi set against a pale blue, monocytic background (BI 6 + and MI 95%). (Images obtained with a 10x ocular lens and a 100x oil-immersion objective lens, yielding an effective magnification of 1,000x). **(B)** SSS stained with the modified ZN method 1 month after initiation of RO therapy, revealing multiple fragmented and granular AFB (BI 6 + and MI 0). (Images obtained with a 10x ocular lens and a 100x oil-immersion objective lens, yielding an effective magnification of 1,000x).

Based on the history and examination, a provisional diagnosis of nodular histoid leprosy was made. An SSS obtained from both ear lobes, eyebrows, and nodules over the extensor surfaces of the forearms (elbows) were stained with the modified Ziehl-Neelsen technique (Table 1). These revealed multiple characteristic long, slender, needle-like acid-fast bacilli arranged as loose clumps, alongside well-formed globi–features overlapping between histoid Hansen’s disease and LL–against a light-blue chronic inflammatory background. The bacteriological index (BI) was 6+, with a morphological index (MI) of 95% (Figure 2A).

**Table 1:** Staining methods used.

Modified Ziehl-Neelsen staining	Time
A	
Air-dry the smear completely on a slide rack.	5 min
Heat-fix by passing (smear side up) once through a clean flame until just warm.	3–5 sec
Primary stain (carbol fuchsin) Flood the entire smear with carbol fuchsin. Gently steam-heat over a flame until uniform steam appears (20–30 sec)—avoid boiling or drying out. Let stain sit at room temperature for 5 min.	20–30 sec heat + 5 min.
No-heat alternative method (The quality of the acid-fast bacilli can be compromised by uncontrolled heating. Therefore, an alternate method can be considered) Cover with fresh carbol fuchsin for 20 min, adding more at 10 min if it begins to dry.	20 min
Decolourise with 5% sulphuric acid until the smear appears very faint pink Wash immediately with a gentle stream of tap water to halt decolourisation.	3–5 sec
Counterstain by flooding smear with methylene blue. Rinse gently with water and air-dry completely before microscopy.	10–20 sec
B	
Fite-Faraco staining protocol	
Deparaffinise 4–5 μm tissue sections in xylene-peanut oil (1:1 solution), twice. Rinse gently under running water.	10–12 min
Primary stain with room-temperature carbol fuchsin. Rinse gently with distilled water.	30 min
Decolorise with acid-alcohol solution (5% sulphuric acid, 25% ethanol) until sections turn pale pink and no stain runoff is visible. Rinse with distilled water.	1 min
Counterstain with Harris haematoxylin (or 0.1% methylene blue for 30 sec). Rinse with water.	3 min
Mount with Dibutyl-phthalate Polystyrene Xylene (DPX) resin after air-drying.	-

**Figure 3:**
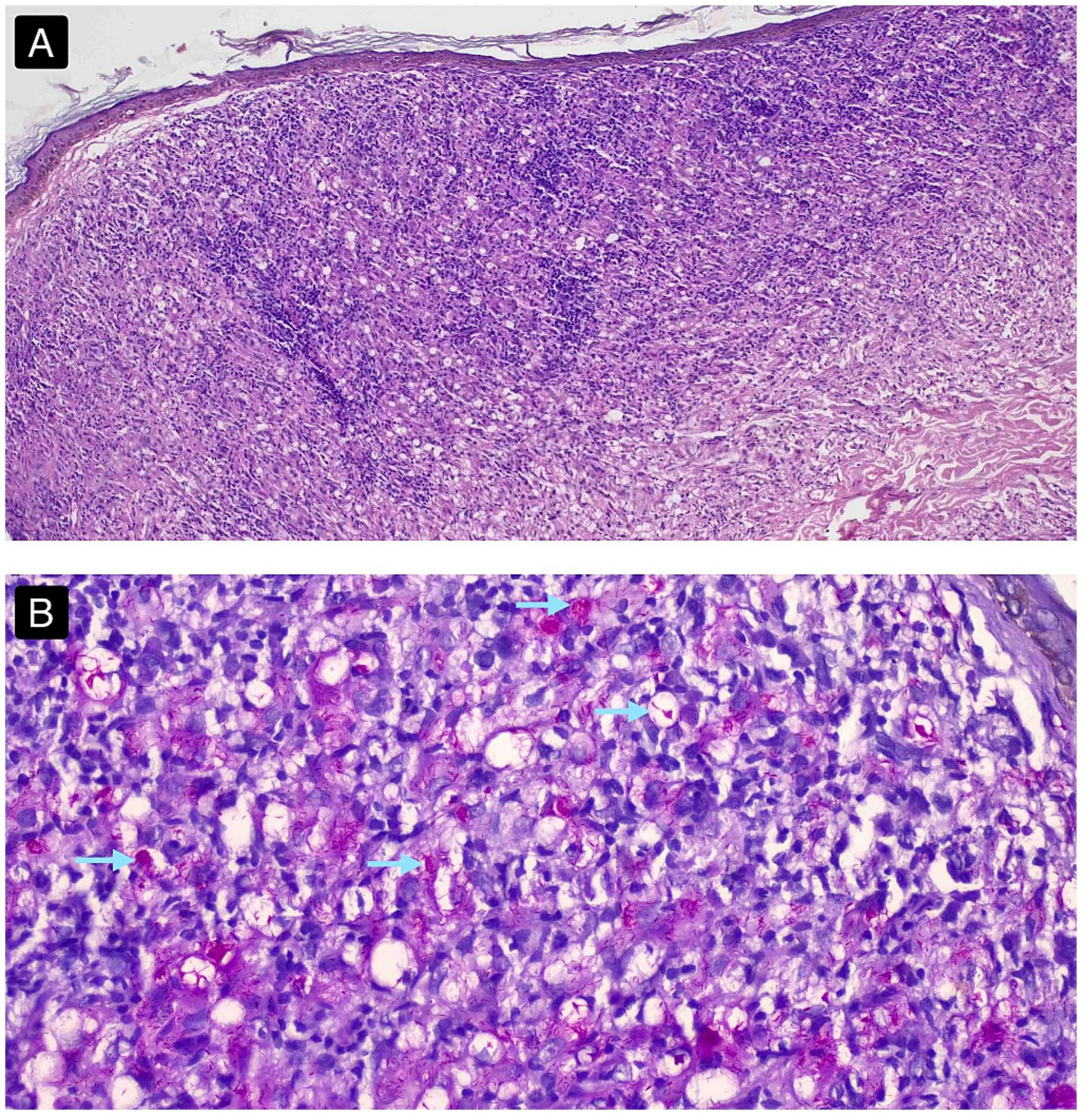
**(A)** H&E-stained section of skin biopsy from a nodule, showing epidermal thinning, multiple, ill-defined granulomas consisting of dense and diffuse infiltration of ill-defined foamy macrophages and histiocytes in the reticular dermis impinging upon the papillary dermis and epidermis, leading to minimal or absence of the grenz zone in the sub-epidermal area, suggestive of lepromatous leprosy. (Images obtained with a 10x ocular lens and a 20x objective lens, yielding an effective magnification of 200x). **(B)** Section stained with the Fite-Faraco method, demonstrating pink-staining slender acid-fast bacilli within foamy macrophages in well-formed globi (blue arrows) and a few extracellular ill-defined bacillary clumps. Note the diffuse infiltration of foamy macrophages with no clear demarcation. (Images obtained with a 10x ocular lens and a 40x objective lens, yielding an effective magnification of 400x).

A 4-mm punch biopsy taken from a nodule on the back revealed diffuse infiltration of foamy macrophages forming ill-defined granulomas within the reticular dermis. These poorly formed granulomas extended upward, obliterating the papillary dermis and impinging on the overlying epidermis, resulting in effacement of the dermal papillae and thinning of the epidermis with minimal to no Grenz-zone of clearance (Figure 3A). Special staining by the Fite-Faraco method (Table 1) confirmed numerous slender acid-fast bacilli arranged in intracellular globi, along with a few extracellular, poorly defined bacillary clumps (Figure 3B), these findings established the diagnosis of LL with a histoid-like clinical profile and bacterial morphology and a close differential diagnosis of Histoid leprosy was made due to the overlapping clinical SSS findings.

To rapidly reduce infectivity, the patient was initially treated with a 28-day course of 600 mg of rifampicin taken on an empty stomach and 400 mg of ofloxacin (RO therapy) while being on the constant lookout for signs of lepra reactions. Following this regimen, a repeat SSS (performed on day 28) on all the previous sites revealed that the acid-fast bacilli appeared fragmented and granular (BI 6 + and MI 0) (Figure 2B), indicating effective bactericidal activity. Thereafter, the patient was commenced on the MB-MDT as per the NELP guidelines. The patient is currently under regular follow-up in liaison with the local healthcare provider (HCP) and the tertiary hospital. In the 5-month follow-up, the patient had significant clinical improvement with no signs of lepra reactions.

## Discussion

Histoid leprosy is recognised as a distinct variant of lepromatous leprosy with a unique clinical and histopathologic profile. The nodular lesions–termed histoid lepromas–can be mistaken for benign dermatological conditions; however, they harbour high bacillary loads, rendering the affected individuals highly infectious (10, 11). Interestingly, despite the high bacillary load, the incidence of lepra reactions in histoid Hansen’s disease is significantly lower compared to classic LL (12). Histopathologically, the cardinal feature of histoid lepromas, is the presence of spindle-shaped histiocytes arranged in a storiform pattern. These cells appear to concentrate numerous acid-fast bacilli in the centre, suggesting a relatively enhanced cell-mediated immune response in MB leprosy.

Ofloxacin is bactericidal to *M. leprae* and has been shown to eliminate ≥ 99% of viable bacilli after 2 weeks, 99.9% after 22 days and 99.99% of the bacillary load when administered daily for 4 weeks, resulting in significant clinical improvement (13–15). A double-blinded randomised control trial (RCT) conducted by Balagon et al. concluded that a FDC of 600 mg of rifampicin and 400mg of ofloxacin taken for four weeks, followed by 5 months of placebo, was comparable to 6 months of MB-MDT in paucibacillary (PB) leprosy, thereby highlighting its therapeutic efficacy, However, this study focused on PB leprosy with low bacillary loads and is not directly applicable to multibacillary (MB) cases (16).

A single dose of oral rifampicin reduces viable *M. leprae* bacilli by 92.1%, and a single dose of rifampicin (600 mg), ofloxacin (400 mg), and minocycline (100 mg) (ROM) serves as effective prophylaxis, though efficacy varies across populations. However, this regimen is suboptimal for therapeutic use due to emerging resistance to rifampicin and ofloxacin. Consequently, single-dose regimens are avoided in clinical practice to prevent further risk of resistance (13, 17).

However, a prospective observational study by Ji et al. demonstrated that, although 85% of multibacillary (MB) leprosy patients treated with a four-week course of RO therapy showed clinical improvement and almost all patients showed sustained MI improvements in SSS 2 years post-treatment, there was an overall relapse rate of 10% (five relapses among 51 cases over 173 patient-years of follow-up). Thus, while 4 weeks of RO therapy is effective in rapidly reducing the bacillary load, its high relapse rates preclude its use as a sole therapy in MB leprosy (18). Instead, an initial 28-day course of RO therapy followed by the commencement of MB-MDT may be beneficial in treating MB leprosy in high-burden and rural areas, with the initial 4 weeks aimed at reducing the infectivity and the subsequent 12 months focused on eradication of the infection (persistors) and preventing relapse.

In a retrospective cohort study by Nascimento et al., among a total of 1,059 leprosy cases reported during the study period, 126 (11.86%)were identified as relapses. Of these relapse cases, 109 (86.5%) had been initially treated with MB-MDT. Within this subgroup, 51 cases (40.5% of total relapse cases) received 12 months of MB-MDT, while 44 cases (34.9%) underwent a 24-month regimen. Notably, 11 cases were found to harbour resistant strains–8 exhibiting rifampicin resistance and 3 showcasing resistance to both rifampicin and dapsone (19). We hypothesise initiating the treatment with a dual regimen comprising two potent bactericidal agents for the initial 28 days, followed by the standard 12-month MB-MDT, may prove beneficial in high-burden settings by rapidly reducing the bacillary load and potentially preventing the emergence of drug resistance.

Rifampicin and ofloxacin are both bactericidal agents. A single dose of RO would have had a significant bactericidal effect; however, in this case, the initial use of the RO regimen was aimed at rapidly reducing the bacillary load and transmissibility–a promising strategy to decrease infectivity in MB leprosy cases. An additional rationale for initiating such an aggressive treatment was the patient’s residence in a rural and remote area, where ensuring substantial and early bacillary clearance was critical given the limited access to follow-up care. The subsequent observation of fragmented acid-fast bacilli on repeat SSS further supports the effectiveness of this short-course therapy in reducing bacterial viability before initiating long-term MB-MDT. It is also important to note that rapid bacillary death increases the risk of lepra reactions by the rapid release of bacillary antigens into the bloodstream. A clinical trial conducted in Vietnam by Khang et al. demonstrated that although the overall incidence of lepra reactions did not differ among various regimes for MB cases, the severity of erythema nodosum leprosum (ENL) reaction was significantly higher in the group treated with only a 28-day course of RO–likely attributable to the absence of clofazimine, which appears to mitigate the severity of such reactions (20). Therefore, strict vigilance is necessary, not only for ensuring treatment compliance but also for early detection and management of lepra reactions in cases with high bacillary loads (Table 2).

**Table 2:** Timeline of events.

Sequential events of case	Interpretation with relevant data
Case presentation (symptoms and brief history; 09/12/2024)	A 50-year-old Indian male presented with gradually progressive nodulo-ulcerative lesions distributed across the body over the past 6 months. He also reported frequent epistaxis, madarosis and weakness of the small muscles of the hands and feet.
Clinical examination (09/12/2024)	Multiple, well-defined, shiny, skin-coloured nodules–with a few ill-defined ulcers–were present over the face, external ears, trunk, back and buttocks.
Baseline SSS (09/12/2024)	Multiple viable acid-fast bacilli observed to be arranged in numerous globi against a chronic inflammatory background (BI—6 + and MI—95%).
Biopsy (Reported 1 week later; 16/12/2024)	Multiple foamy macrophages infiltrating the papillary and reticular dermis seen. Fite-Faraco staining demonstrated multiple globi within these macrophages.
Treatment initiation (with RO therapy; 16/12/2024)	Patient was prescribed 600 mg of rifampicin taken on empty stomach and 400 mg ofloxacin after breakfast daily for 28 days.
SSS 28-days post RO therapy (14/01/2025)	Multiple fragmented and granular acid-fast bacilli were observed (BI—6 + and MI—0%).
Initiation of MB-MDT (according to NELP guidelines; 15/01/2025)	Patient was subsequently started on standard MB-MDT (No signs of lepra reactions).
Outcome and follow-up (15/05/2025)	At the 5th month following the initiation of MB-MDT, the patient exhibited a significant reduction in skin lesions with no signs of lepra reactions (BI was still 6 + and MI—0%).

The absence of lepra reactions despite the use of this aggressive therapy and the rapid MI decline at 28 days and throughout the 5-month follow-up may be attributed to clofazimine in the MB-MDT regimen or immunomodulatory properties inherent to the overlapping or developing histoid-like features. However, it is important to note that despite the current absence of lepra reactions, this does not mitigate the risk of future occurrences. Initiating such aggressive treatment approaches warrant close monitoring by HCPs to promptly identify any signs of lepra reactions. Additionally, patient counselling and education are essential to ensure awareness of both early and late manifestations of lepra reactions (21).

It is noteworthy that such high bacillary loads are uncommon in the current era of leprosy elimination, where early detection and prompt treatment have significantly reduced their incidence. While the patient–in this case–reported symptom progression over 6 months, *M. leprae*’s prolonged generation time (~14 days) and the observed BI 6 + suggest a chronic infection likely spanning over several years. This delay underscores systemic challenges in rural healthcare access and highlights the need for community-level awareness programmes. Moreover, histoid Hansen’s disease has been associated with resistant strains of *M. leprae,* often resulting from dapsone monotherapy. This variant may present *de novo* due to transmission of resistant bacilli in the community or arise from incomplete or inadequate treatment, thereby complicating disease management and contributing to ongoing transmission (22).

It is important to note that while multibacillary leprosy cases may exhibit a high MI, values typically range between 20 and 25% and rarely exceed 50%, even in advanced, untreated LL. An MI exceeding 95% is exceptionally rare and unprecedented. Such cases necessitate sequential SSS over consecutive visits to accurately monitor MI decline post-treatment initiation. However, the patient’s remote rural residence posed logistical challenges for the hospital team to conduct these follow-ups at regular intervals.

The MI values reported in this case were derived from smears obtained from the earlobes, eyebrows and elbow nodules, which were independently reviewed twice by trained staff. The rapid MI decline (from 95 to 0%) likely reflects both the bacilli’s drug sensitivity and the intensive 28-day RO regimen. While the smears were analysed by trained and qualified personnel, it is critical to acknowledge that the patient’s remote rural residence limited regular follow-up, potentially introducing bias in MI assessment due to inconsistent monitoring intervals.

In a retrospective observational study conducted by Patil et al. of 160 MB leprosy cases found that only 14 (8.75%) exhibited an MI > 50% (23). Another study by Kalla et al., conducted in a reportedly non-endemic region of India, reported MI values ranging from 41–90% (mean: 69.5%) in 25 biopsy-proven histoid leprosy cases, based on smears from lepromas (24).

The 95% MI observed in our case likely reflects prolonged, untreated infection with the Hansen’s bacilli, resulting in a predominance of viable, solid-staining bacilli. This finding highlights the inherent subjectivity of MI assessment and underscores the need for advanced molecular methods—such as the viability assay described by Lenz et al.—to objectively evaluate bacterial viability (25).

Our case exhibited clinical features suggestive of histoid leprosy, including shiny, keloid-like nodules with occasional umbilication that later progressed to rupture and necrosis. However, SSS revealed overlapping characteristics: slender needle-like bacilli consistent with the histoid variant coexisted with the characteristic, well-formed globi, consistent with LL. Histopathological examination further supported the diagnosis—while classic storiform histoid habitus was absent, diffuse infiltration by foamy macrophages aligned more closely with LL. Based on these findings, the diagnosis of LL was established, with histoid Hansen’s disease considered as a close differential.

We speculate and hypothesise that this case may represent LL undergoing bacillary and immunological transformation towards a histoid morphology, reflecting the dynamic nature of *M. leprae* in chronic infections. This underscores the importance of strict adherence to treatment protocols and robust surveillance mechanisms to detect and manage such cases at an early stage.

While this case provides promising preliminary evidence, it is important to acknowledge several limitations. The findings are based on a single patient, which inherently limits the generalizability of the results. Additionally, the short follow-up period (5 months) after the initial RO regimen, while sufficient to observe early bactericidal effects, does not fully capture long-term outcomes such as relapse rates and late-onset lepra reactions, which can occur months and years post-treatment. Resource constraints in rural settings precluded advanced testing (e.g., PCR) for drug resistance profiling as well as comprehensive and sequential BI and MI assessment. Nevertheless, the fragmented and granular bacilli observed after RO therapy suggested retained treatment susceptibility, supporting the regimen’s bactericidal efficacy in this case.

Further studies with larger cohorts and controlled clinical trial designs coupled with drug-resistance monitoring are necessary to validate these observations and to determine the optimal duration and combination of bactericidal agents. Long-term follow-up will be needed to assess not only the efficacy but also the safety profile of this two-step treatment strategy, particularly concerning the risk of drug resistance and the management of lepra reactions.

## Conclusion

This case of nodular histoid-like LL in a 50-year-old male underscores the public health and therapeutic challenges posed by MB leprosy in the post-elimination era. A two-step treatment strategy–initially reducing bacillary load with a 28-day course of RO regimen followed by MB-MDT–not only demonstrates rapid infectivity reduction but also offers a feasible approach for improved management in high-burden settings with probable lower relapse rates and decreases the possibility of developing drug resistance. Further studies with larger cohorts are warranted to validate and potentially establish its role in current clinical practice.

## Data Availability

The original contributions presented in the study are included in the article/supplementary material, further inquiries can be directed to the corresponding author.
